# Dietary Soy Isoflavones Promote Feminization and Enhance Growth of Juvenile Japanese Eel (*Anguilla japonica*)

**DOI:** 10.3390/ani15172513

**Published:** 2025-08-26

**Authors:** Hae Seung Jeong, Seong Don Hwang, Kyoung Mi Won, Ju-ae Hwang

**Affiliations:** 1 Advanced Aquaculture Research Center, National Institute of Fisheries Science, Changwon 51688, Republic of Korea; jhs0322@korea.kr; 2 Division of Convergence on Marine Science, Korea Maritime and Ocean University, Busan 49112, Republic of Korea; sdhwang@kmou.ac.kr; 3 Aquaculture Research Division, National Institute of Fisheries Science, Busan 46083, Republic of Korea; kyoungmiwon@korea.kr

**Keywords:** feminization, growth performance, Japanese eel, soy isoflavone

## Abstract

The Japanese eel (*Anguilla japonica*) is a valuable aquaculture species in East Asia. However, sex differentiation under culture conditions is often male-biased, complicating the production of female broodstock. This study evaluated the effects of dietary soy isoflavone (SI) supplementation on growth performance, sex differentiation, and sex-specific gene expression in juvenile eels. Juvenile eels (initial mean weight: 1.25 ± 0.02 g) were randomly assigned to 15 tanks, each containing 100 g eels, in a 30-week feeding trial. The control diet (SI0) contained 75% jack mackerel meal. The graded levels (2.5, 5, 7.5, and 10%) of α-Starch were included at the expense of SI, referred to as SI0, SI2.5, SI5, SI7.5, and SI10 diets, respectively. Fish were hand-fed to satiation in triplicate groups of each diet, twice daily for 30 weeks. The SI2.5 and SI5 groups exhibited significantly enhanced final weights and specific growth rates compared to those in the control group (SI0). Histological analysis revealed the presence of differentiating gonads with oogonial proliferation and previtellogenesis in the SI-treated groups, particularly at the 2.5% and 5% inclusion levels. Additionally, the expression of key female-related genes (*vasa*, *cyp19a1a*, *foxl2a*, *zp3*, and *zar1*) was significantly upregulated in these groups. In contrast, relatively high SI levels (7.5% and 10%) did not provide any further benefits. These results suggest that low-dose supplementation of dietary SI can enhance feminization and promote growth in juvenile eels, thus offering a potential natural alternative to synthetic hormone treatment for broodstock development.

## Simple Summary

The Japanese eel (*Anguilla japonica*) is a commercially vital aquaculture species in East Asia. However, the proportion of females required for breeding is often low under farming conditions because of male-biased sex differentiation. This study investigated the effects of dietary soy isoflavones as a natural alternative to hormonal treatment. Soy isoflavone supplementation improved growth performance and feminization in juvenile eels. Therefore, soy isoflavone supplementation could be a sustainable strategy for supporting broodstock development in eel aquaculture.

## 1. Introduction

The Japanese eel (*Anguilla japonica*) is commonly cultured and consumed as a luxury food in East Asian countries, such as China, Japan, and the Republic of Korea, because of its high economic and nutritional value [1,2,3,4]. Japanese eels are the most commercially important inland aquaculture species in the Republic of Korea. The total aquaculture production of eels in the Republic of Korea is approximately 16,058 tons, and the production value of farmed eels is approximately 514 billion KRW, accounting for 82% of the total inland fish aquaculture production value in 2024 [5].

The glass eel used in its aquaculture is obtained from estuaries. However, the arrival of glass eels has dramatically decreased since the 1970s, which has become a serious problem in East Asian countries [6,7,8]. Therefore, establishing a method for the seed production of eels is necessary. The artificial production of glass eels is very difficult, and achieving a stable supply of high-quality eggs is a major concern in seed production. Egg quality, in terms of the resulting fertilization, hatching, and survival rates, is highly variable among eggs from each eel [9]. Therefore, improved techniques are necessary for producing high-quality eggs to enable the mass production of juvenile eels. The sex ratio of eels has become more biased towards males due to cultural environments rather than the research field [10,11,12]. However, the mechanism underlying male bias in cultured eels remains unidentified [7]. Therefore, a sustainable supply of female eels under culture conditions is essential for the successful seed production of Japanese eels.

Currently, the oral administration of 17β-estradiol (E_2_) is used for the artificial seedling production of eels. [10,13,14]. Juvenile eels (*A. japonica*) fed on a diet supplemented with 25, 50, and 75 mg/kg E_2_ have shown feminization [10]. To induce feminization, the juvenile eels are fed synthetic hormones for 4 months and then intensively reared in an isolated culture facility [15]. However, this feminization method causes low egg quality, high economic costs, and time-consuming processes [16].

Soybeans, generally used as a major source of plant proteins for farmed fish, contain phytoestrogens called soy isoflavone (SI) [17,18]. Phytoestrogens are plant-derived compounds that mimic estrogen and its activities [14,19,20]. Phytoestrogens can bind to steroid-binding proteins and the estrogen receptors of target cells [21]. One of the phytoestrogens used in aquaculture is SI, which is structurally comparable to E_2_ and applies numerous estrogen-like biological effects to the health, growth, and reproductive functions of fish [21]. SIs are non-steroidal phenolic secondary metabolites produced during the growth of soybeans [22]. SIs possess numerous physiological functions and provide a wide range of biological effects, such as antioxidant and anti-inflammatory effects, cancer-cell-growth inhibition, and osteoporosis prevention.

SIs can potentially induce feminization in various species of fish, such as the Japanese eel (*Anguilla japonica*), European eel (*Anguilla Anguilla*), Southern flounder (*Paralichthys lethostigma*), Nile tilapia (*Oreochromis niloticus*), Japanese Medaka (*Oryzias latipes*), Russian sturgeon (*Acipenser gueldenstaedtii*), and rainbow trout (*Oncorhynchus mykiss*) [7,14,17,23,24,25,26]. In particular, Japanese eels fed soybean isoflavones (SI) at a low dose (2 g/kg feed) resulted in 9.3% females, whereas those fed 10 and 50 g/kg feed SI produced 91.6% and 96.6% females, respectively [7].

However, to our knowledge, no study has developed experimental diets containing SIs for Japanese eels to assess their effects on growth performance and feminization efficiency. Therefore, in this study, we investigated the effects of dietary SI on growth performance and feminization by examining the expression of sex-related genes and histological analysis in the gonad of juvenile Japanese eels.

## 2. Materials and Methods

### 2.1. Preparation of the Experimental Eels and Conditions

Glass eels (0.11 ± 0.01 g, n = 20) were bought from a private hatchery (Hampyeong-gun, Jeollanam-do, Republic of Korea). The glass eels were acclimated to the experimental rearing conditions for 1 week and fed two types of commercial feed (Nosan Corporation, Minatomirai, Yokohama, Kanagawa, Japan) for two months. After acclimatization, when the glass eels became elvers, they (initial weight, 1.28 g) were randomly assigned to a total of 15 50 L rectangular glass tanks (water volume, 40 L), with triplicate tanks for each of the five experimental diets (100 g per tank). The rearing tanks were filled with underground water filtered through a sand filter and sterilized with a UV lamp, and proper aeration was provided. The water of each tank was changed twice daily, after 30 min of feed supply, and the eels were maintained in the dark throughout the experiment. To maintain water quality, excreta were removed by siphoning, and dead fish were also removed daily. The temperature, dissolved oxygen (DO), pH, total ammonia nitrogen, and nitrite nitrogen of the water were 26.84–26.89 °C, 6.62–6.68 mg/L, 7.20–7.28, 0.29–0.43 mg/L, and 0.21–0.35 mg/L, respectively.

### 2.2. Preparation of the Experimental Diets

The five experimental diets used in this study are presented in Table 1. The control diet (SI0) contained 75% fishmeal (jackmackerel meal) as the protein source and 22% α-starch as the carbohydrate source. The crude protein and lipid levels in SI0 were 55.5 and 6.97%, respectively. SI (2.5, 5, 7.5, and 10%) was included instead of the same amount of α-starch to maintain a balanced diet composition without altering the levels of protein and lipid, which are critical for eel growth, and the diets were referred to as SI2.5, SI5, SI7.5, and SI10, respectively. The content of SIs (Miraebiotech Corporation, Pochen-si, Gyeonggi-do, Republic of Korea) used in these diets was 40 mg/g. The feed ingredients were thoroughly mixed, and water was added at a ratio of 1:1 to ensure the consistency of the mixture before the feed supply.

### 2.3. Sample Collection

After 30-week feeding trials, all eels in the tank were starved for 24 h and then anesthetized with 500 ppm tricaine methanesulfonate (MS-222, Sigma-Aldrich, St. Louis, MO, USA). All live eels from each tank were counted and collectively weighed to determine the effects of the experimental diets on the survival rate, weight gain, and specific growth rate (SGR). Fifteen eels per tank were randomly sampled to calculate the condition factor (CF), viscerosomatic index (VSI), and hepatosomatic index (HSI). To determine the final chemical composition of the body, five eels from each tank were randomly frozen. Moreover, five eels were randomly chosen for blood sampling using a 1 mL heparin-treated syringe. Plasma samples were collected as separate aliquots following centrifugation at 7500 rpm for 15 min at 4 °C and stored at −80 °C until analysis [27].

### 2.4. Chemical Composition of the Diets and Whole Body of Eels

The chemical compositions of the experimental diets and pooled whole bodies of eels were analyzed following the method of AOAC [28] and Jeong et al. [29].

### 2.5. RNA Extraction and Quantitative Real-Time PCR Analysis

The gonads of eels in each tank (n = 10) were sampled after the experimental period. Total RNA was extracted from gonads using a RNeasy Plus Mini Kit (Qiagen, Germany), according to the manufacturer’s instructions. Total RNA was reverse-transcribed using an M-MLV cDNA Synthesis Kit (Enzynomics, Daejeon, Republic of Korea). Quantitative real-time polymerase chain reaction (PCR) was carried out with a TOPreal SYBR Green qPCR PreMIX (Enzynomics) using the cDNA templates and primers targeting sex-differentiation genes (Table 2) on a CronoSTAR 96 Real-Time PCR System (Clontech, Takara Bio Inc., Shiga, Japan) [27]. The PCR conditions were as follows: 40 cycles of denaturation at 95 °C for 10 s, and annealing and extension at 60 °C for 45 s. Gene expression was analyzed using the 2^−ΔΔCt^ method and normalized with respect to an endogenous reference *β-actin*.

### 2.6. Isoflavone Analysis of the Experimental Diets

The amounts of isoflavone, including daidzin, daidzein, genistin, genistein, and glycitein, in the experimental diets were quantified using liquid chromatography–tandem mass spectrometry (1290 Infinity II LC system, Agilent technologies, Santa Clara, CA, USA and 6470 triple Quadrupole LC/MS, Santa Clara, CA, USA). Data were acquired using the Agilent MassHunter Workstation software (LC/MS Data Acquisition for 6470 Series Triple Quadrupole v.B.08.02) and quantified using the Agilent MassHunter Workstation software (Quantitative Analysis for QQQ v.B.08.00).

### 2.7. Histological Analysis

The time of sacrifice was defined as the time when the gonads were differentiated according to previously described classification methods [30], and the experiment ended when an individual of 20–30 cm was observed. After collecting blood, the gonads and body segments were dissected. Separated gonads were fixed overnight in 10% neutral-buffered formalin and embedded in paraffin. Sections (5 µm) were stained with hematoxylin and eosin (BBC Biochemical, Mount Vernon, WA, USA) and observed using an optical microscope (AxioCam MR; Carl Zeiss, Jena, Germany) to determine the sex ratio and stages of gonad development. Sex differentiation and determination were confirmed, as previously described [30].

### 2.8. Calculation and Statistical Analysis

Growth performance was calculated as follows:

Survival (%) = (number of eels at the end of the trial/number of eels at the start of the trial) × 100,(1)

Weight gain (WG; g/fish) = final body weight − initial body weight,(2)

SGR (%/day) = [(ln final weight of fish − ln initial weight of fish)/days of trial] × 100,(3)

CF (g/cm^3^) = body weight (g) × 100/total length (cm)^3^, (4)

VSI (%) = viscera weight × 100/body weight,(5)

HSI (%) = liver weight × 100/body weight.(6)

The Shapiro–Wilk test was used to examine the normality of distribution, and the Levene’s test was used to evaluate the homogeneity of variance among treatments. Prior to statistical analysis, percentage data were transformed using an arcsine square-root method to stabilize variances. One-way ANOVA and Tukey’s honest significant difference test were used to analyze significant differences among the groups. All analyses were performed using SPSS 24 (SPSS Inc., Chicago, IL, USA).

## 3. Results

### 3.1. Growth Performance of Eels

The growth parameters of eels are presented in Table 3. The survival rates were 85.4–95.9%, showing no differences among the groups (*p* > 0.05). The final weight, WG, and SGR of eels fed SI2.5 were significantly higher than those of the eels fed SI0 (*p* < 0.05); however, the values did not differ from those of the SI5, SI7.5, and SI10 groups (*p* > 0.05). CF and VSI were not significantly affected by dietary SI supplementation (*p* > 0.05). the HSI of eels fed SI2.5 was significantly higher than the eels fed SI0 (*p* < 0.05); however, it did not differ from the eels fed SI5, SI7.5, and SI10 (*p* > 0.05).

### 3.2. Chemical Composition of the Whole Body of Eels

Table 4 presents chemical compositions of the whole bodies of eels. Whole-body crude protein content in eels fed SI2.5 was significantly higher than that in eels fed SI7.5, SI10, and SI0 (*p* < 0.05); however, it did not significantly differ from the eels fed SI5. The crude lipid content in the eels fed SI0 was significantly higher than in the eels fed SI2.5 and SI5 (*p* < 0.05); however, it did not significantly differ from the eels fed SI7.5 and SI10. Moisture and ash contents in the whole body were 68.6–69.9% and 2.0–2.3%, respectively. Dietary SI did not affect the moisture and ash contents in the whole body of the eels.

### 3.3. Sex Determination Ratio of Eels and Histological Analysis

**Figure 1:**
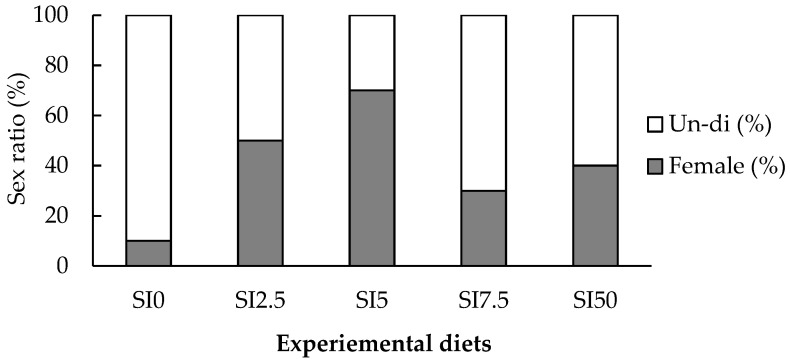
Sex-differentiation ratio in eels fed experimental diets with different soy isoflavone contents for 30 weeks (n = 15). Un-di, Undifferentiated.

Sexual differentiation induced by SI treatment was divided into two stages (undifferentiated and differentiating for early females with PGC and OP stages) to compare the sex ratio (Figure 1). Histological analysis of the SI-treated groups revealed that the average length was 22–25 cm. When compared with the control group, all SI-treated eels were induced to become females (26.66–66.67%), starting from the SI2.5 group (46.67%, 25.3 ± 1.02 cm body length). The feminization ratio was highest at 66.67% in the SI5 group (25.5 ± 0.66 cm body length). The SI7.5 (22.9 ± 1.08 cm body length) and SI10 (22.6 ± 0.44 cm body length) groups showed feminization ratios of 33.33% and 26.66%, respectively, which were lower than that of the SI2.5 group. More females were observed in the SI5 group than in the SI2.5 group, and the OP stage was dominant in the differentiation stage. In the SI7.5 and SI10 groups, some individuals presumed to be females were observed by histological analysis, and PGC and OP stages were observed in matured individuals.

**Figure 2:**
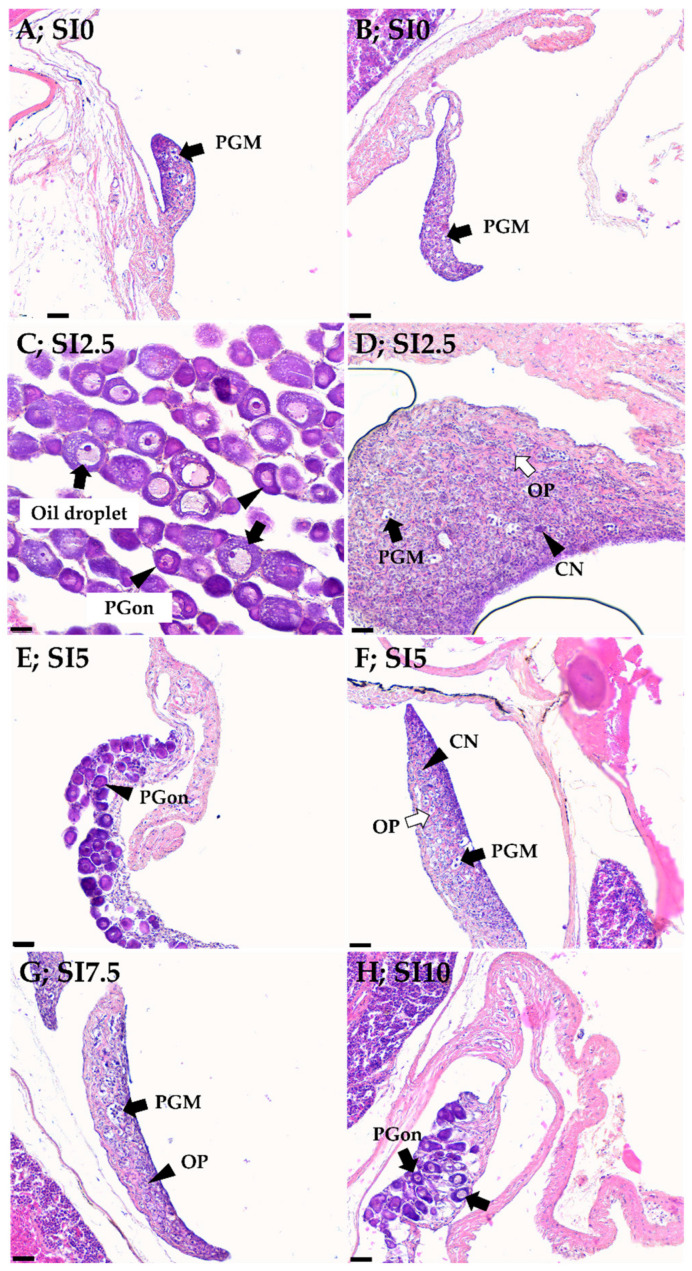
Histological observation of gonads in Japanese eel treated with PBS and isoflavone. (**A**): primordial germ cells (arrows) in an undifferentiated state in control group(SI0); (**B**): primordial germ cells (arrows) in an undifferentiated state in control group(SI0); (**C**): Numerous oil droplet stage oocytes (arrows) and a few one-nucleolus (PGon) stage oocytes (arrowheads) at the the primary growth (Previtellogenesis, PG) stage in the group fed SI2.5; (**D**): A few primordial germ cells (black arrows) in an undifferentiated state, numerous OP stage oocytes (white arrows), and developing chromatin nucleolus (CN) stage oocytes (arrowheads) in the group fed SI2.5; (**E**): Numerous PGon stage oocytes (arrowheads) at the PG stage in the group fed SI5; (**F**): A few primordial germ cells (black arrows) in an undifferentiated state, OP stage oocytes (white arrows), and developing CN stage oocytes (arrowheads) in the group fed SI5; (**G**): primordial germ cells (arrows) in an undifferentiated state (arrows) and oogonial proliferation (OP) stage oocytes (arrowheads) in the group fed SI7.5; (**H**): PGon stage (arrowheads) at the PG stage in the group fed SI10. Scale bar; 30 µm.

Histological analysis (n = 15) was performed after 30 weeks of a feeding trial to assess the effects of dietary SI on sex determination and the differentiation of eels (Figure 2). In the control group (SI0), almost all of the eels were undifferentiated (93.33%), and no intersexual stage or male-like gonads were identified. Even the gonads of the eels fed SI0 and measuring > 28 cm were undifferentiated. Figure 2B shows a female in the PGC stage; however, most of them were in an undifferentiated gonad state (Figure 2A), and the sex could not be distinguished.

In the SI2.5 group, most eels were in the OP (oogonial proliferation) stage (Figure 2D,F,G; 22–28 cm body length), in which differentiation into females was in progress.

In Figure 2C (42.3 cm body length), fish treated with 2.5% SI were estimated to be females, and the oil droplet stage was dominant in the previtellogenesis stage. However, some individuals showed PGC (primordial germ cell) stages that were indistinguishable between the sexes, similar to those in the control group. Eels treated with 10% SI had a body length of 23.5 cm, and differentiation into females was in progress (Figure 2H).

### 3.4. Expression of Sex-Specific Genes in the Gonads

**Figure 3:**
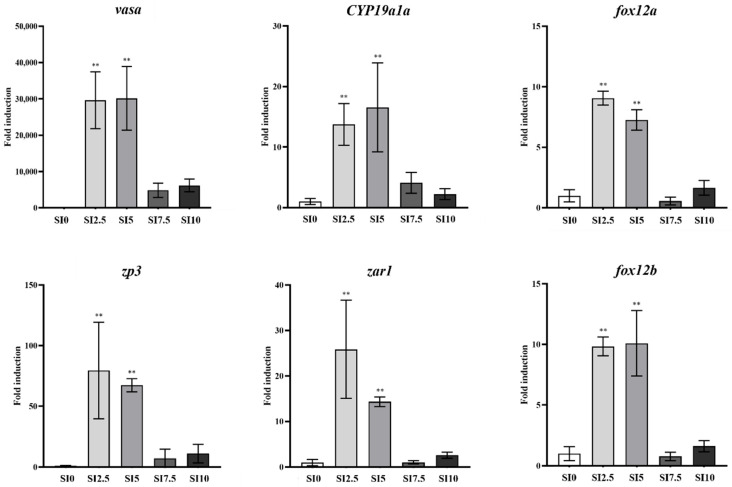
The expression of sex-differentiation genes in the gonads of eels fed experimental diets with different soy isoflavone contents for 30 weeks (n = 30). Data represent mean ± standard deviation. ** *p* < 0.01 between the control and SI-treated groups.

The expression of all sex-differentiation genes was considerably higher in fish of the SI2.5 and SI5 groups than in fish of the SI0 group (Figure 3).

The expression of *vasa* (dead-box protein vasa), *cyp19a1a* (cytochrome P450, family 19, subfamily A, polypeptide 1a), *foxl2a* (Forkhead box L2a), *zp3* (Zona pellucida sperm-binding protein 3), *zar1* (Zygote arrest 1), and *foxl2b* (Forkhead box L2b) in the gonads of the SI2.5 group significantly increased by 29,630.4-fold (*p* < 0.01), 13.7-fold (*p* < 0.01), 9.1-fold (*p* < 0.01), 79.5-fold (*p* < 0.01), 25.8-fold (*p* < 0.01), and 9.8-fold (*p* < 0.01), respectively, compared to that of the SI0 group. In the SI5 group, the expression of these genes increased 30,158.3-fold (*p* < 0.01), 16.6-fold (*p* < 0.01), 7.3-fold (*p* < 0.01), 67.3-fold (*p* < 0.01), 14.3-fold (*p* < 0.01), and 10.1-fold (*p* < 0.01), respectively, compared to that in the SI0 group. In contrast, the SI7.5 and SI10 groups did not show significant differences in the expression of these genes compared to that of the SI0 group.

## 4. Discussion

Under culture conditions, the sex ratio of eels becomes relatively biased towards males [10,11,12]. Female eels gain higher body weight and have better commercial value than males; however, they are also necessary for the successful production of artificial seedlings. Therefore, increasing the percentage of females would be beneficial for eel culture. The use of synthetic steroid hormones, such as E_2_, in fish farming is illegal and compromises the safety of aquaculture products [15,16]. Although E_2_ has a strong female-inducing function, the problem of water purification treatment cost to remove E_2_, and the growth inhibition [30,32] caused by E_2_ treatment, shows negative results regarding the E_2_ hormone. Therefore, feminization methods using phytoestrogens, particularly SI, may be suitable for eel farming.

Several studies have reported the feminization effects of SI in various fish species [7,14,17,23,24,25,26]; however, the supplementary effect of SI in formulated diets on the Japanese eel has not been investigated.

In this study, the higher final weight, WG, and SGR of eels fed SI-supplemented diets than the eels fed the control diet revealed that dietary supplementation of SI led to improved growth performance in eels. Similar results have been reported for other fish species, such as olive flounder (*Paralichthys olivaceus*), Atlantic salmon (*Salmo salar* L.), and rainbow trout (*O. mykiss*) [21,33,34]. Juvenile grass carp fed diets containing a 500 mg SI/kg diet showed better growth than fish fed diets without SI [35]. Similarly, the growth performance of golden pompano (*Trachinotus ovatus*) was significantly increased by dietary SI levels up to 40 mg/kg, which decreased with a further increase in dietary SI content, indicating that dietary SI up to a suitable dose may promote fish growth [36]. In contrast, SI has been reported to negatively affect fish growth, such as southern flounder (*P. lethostigma*) and rice field eels (*Monopterus albus*) [23,37]. The varying effects of SI supplementation observed in different studies may be due to varying dietary SI doses or differences in the sensitivity of species.

The highest growth performance was obtained in the SI2.5 group, which considerably decreased as the dietary SI level increased. Relatively high levels of dietary SI reduced α-starch content, which decreased the water stability of the feed, resulting in relatively high leaching in water. This could be a reason for the better growth performance of eels fed diets with lower SI content than the eels fed diets with higher SI content. Improved water stability of formulated diets contributes to elevated growth performance [38,39].

Nutrient composition and nutritional values are associated with fish age and size [40]. The highest crude protein content of the whole body of an eel was obtained in the SI2.5 group, which considerably decreased as the dietary SI level increased. The lowest crude protein content was obtained in the SI0 group. The highest crude lipid content in the whole body was obtained in the SI0 group, followed by those of the SI10, SI7.5, SI5, and SI2.5 groups. The protein contents of some fish species slightly increased or remained relatively stable as the body weight increased [41]. In contrast, it was reported that the chemical composition was not affected by growth performance in olive flounder [29]. In this study, the crude protein contents of eels showed an increasing trend with improved growth performance, whereas the crude lipid contents decreased as growth performance increased. It has been recognized that the chemical composition of fish is affected by several endogenous and exogenous factors [42].

Sex determination and differentiation in yellow eels at the juvenile stage are not well understood; however, the strong induction of feminization (70–97%) by hormonal treatment, such as E2, has been reported [7,14]. The effects of plant extracts, phytoestrogen, and E2 have been reported in European eels [14]. Recently, the feminization effect of SI in Japanese eels has been reported [7]. In this study, we observed that feminization was successfully induced even with a low dose (2.5%) of SI. Soybean treatment for 210 days resulted in 40% and 66% of females in the SI2.5 and SI5 groups, respectively. The gonadal differentiation status of individuals confirmed as females was observed as differentiating or differentiated with previtellogenesis and oogonial proliferation. The SI7.5 and SI10 groups had only 20% and 30% females, respectively. This is consistent with a low ratio (13%) of females in European eels [14] treated with high doses of genistein (20 mg/kg). In contrast, high doses of soybean have led to higher proportions of females in the Japanese eel [7].

In the control group, 6.6% were females; the rest consisted of undifferentiated individuals; no males were identified. This sex ratio was similar to that reported in another study (5.4%) [14]. Environmental factors involved in the sex determination of eels included temperature and density, and high temperature and density affect the sex determination of males in eels [43] and several other fish species [44,45,46]. Eels raised in general farms with high density and high temperature are mostly males. Moreover, eels raised at high densities in the laboratory are mostly males [14]. In this study, the density of eels was higher than that previously reported [7], with 70–80 eels (1.22 g/eel) in a 50 L tank. The female ratio in the control group was 6.6%, and 53% (24/45) of the eels were < 20 cm in length. Therefore, the exact sex ratio could not be confirmed as the eels were in an undifferentiated state, as previously reported [30]. In the study reporting an increase in the proportion of females in the low-density experimental group [14], the temperature of the breeding water was maintained at 24–26 °C, which was lower than that used in the present study (average 26 °C), and the breeding environment was also different. Therefore, future studies should examine the effects of water temperature, density, and breeding for a long period.

The expression of sex-specific genes increased, particularly in SI-treated eels (2.5% and 5%), compared to that in the control. *vasa* was highly expressed in differentiated female eels and turbot [30,47]. *Cyp1a1* and *Foxl2a* play important roles in ovarian differentiation in eels [48]. In the SI2.5 and SI5 groups with a high proportion of female eels, *Cyp1a1* and *Foxl2a* expression was significantly relatively high, similar to that previously reported [7,48]. However, *Cyp1a1* expression was not significantly different between males and females, and *Cyp1a1* was indirectly induced by E_2_ treatment [30]. *Foxl2* directly induces *Cyp1a1* expression in Japanese flounder [49] and Japanese eels [7,48], which is consistent with our experimental results. *Zp3* and *zar1* play important roles in early oocyte development [50]. To date, the expression of these two genes has not been observed in juvenile eels treated with natural extracts for the purpose of feminization. In the present study, along with the expression of other female-specific genes, *Zp3* and *zar1* expression significantly increased according to the SI treatment.

The sex determination period in eels is reported to be between 20 and 30 cm [51]. As documented [30,48], the present results also showed that feminization (TL; 22–25 cm) was induced in eels averaging 10 cm by natural extract treatment. Jeng et al., [30] reported the expression of genes related to sex differentiation in undifferentiated and differentiated states using E_2_ treatment. But most published studies only report correlations between the expression of specific genes, such as *Foxl2* and *Cyp1a1*, after sex differentiation. However, due to biological size issues in eels, such as the small organs and sampling of anatomically indistinguishable gonads in eels between 15 and 30 cm, it is difficult to provide a clear basis for sex determination and differentiation. We have previously reported that ≥90% males are induced after 180 days of MT treatment in eels; in addition, most (80%) immature individuals could be confirmed in the control group [27], similar to the results of the present study. Furthermore, the finding that no males were identified in any group is consistent with reports that differentiation into males proceeds later in larger individuals (>40 cm) than in females [30,52,53]. Therefore, the expression of male-related sex genes was not assessed in the present study. Future studies will extend the observation period to examine the mechanisms of male and female differentiation and compare growth. Our results suggest that *Cyp1a1*, *Foxl2*, *Zp3*, and *zar1* are important for early feminization of yellow eels induced by SI treatment.

## 5. Conclusions

Our findings demonstrate that dietary SI at low inclusion levels (2.5–5%) can effectively promote growth and feminization in juvenile Japanese eels, offering a promising natural alternative to synthetic hormones in aquaculture. Although the present study was conducted at the juvenile stage and thus cannot yet provide direct evidence regarding broodstock maturation, egg quality, or cost and time efficiency compared with the conventional E_2_-based method, these aspects remain critical for commercial application. We therefore acknowledge the practical significance of this issue and will address it in future long-term studies.

## Figures and Tables

**Table 1:** Ingredients, chemical composition, and isoflavone contents of the experimental diets (dry matter basis, %).

	SI0	SI2.5	SI5	SI7.5	SI10
Ingredients (%)					
Jack mackerel meal	75	75	75	75	75
α-Starch	23	20.5	18	15.5	13
Soy isoflavone	0	2.5	5	7.5	10
Vitamin C	0.3	0.3	0.3	0.3	0.3
Vitamin E	0.2	0.2	0.2	0.2	0.2
Vitamin Mix ^1^	0.6	0.6	0.6	0.6	0.6
Mineral Mix ^2^	0.4	0.4	0.4	0.4	0.4
MCP ^3^	0.5	0.5	0.5	0.5	0.5
Chemical composition (%)					
Dry matter	90.8	91.0	91.4	91.8	92.2
Crude protein	55.5	55.5	55.6	55.7	55.7
Crude lipid	6.9	7.0	7.1	7.3	7.4
Ash	10.3	10.4	10.4	10.5	10.5
Isoflavone contents (%)					
Daidzin		0.182	0.306	0.470	0.638
Daidzein		0.807	1.582	1.811	2.123
Genistin		0.041	0.070	0.104	0.140
Genistein		0.001	0.001	0.002	0.002
Glycitein		0.001	0.001	0.002	0.002

^1^ Vitamin premix contained the following components (g/kg in diets): ascorbic acid, 300; dl-calcium pantothenate, 150; choline bitartrate, 3000; inositol, 150; menadione, 6; niacin, 150; pyridoxine · HCl, 15; riboflavin, 30; thiamine mononitrate, 15; dl-α-tocopherol acetate, 201; retinyl acetate, 6; biotin, 1.5; folic acid, 5.4; and cobalamin, 0.06. ^2^ Mineral premix contained the following components (g/kg in diets): NaCl, 437.4; MgSO_4_·7H_2_O, 1379.8; ZnSO_4_·7H_2_O, 226.4; Fe-citrate, 299; MnSO_4_, 0.016; FeSO_4_, 0.0378; CuSO_4_, 0.00033; calcium iodate, 0.0006; MgO, 0.00135; and NaSeO_3_, 0.00025. ^3^ Monocalcium phosphate.

**Table 2:** Specific primers used for real-time quantitative polymerase chain reaction.

Genes	Forward (5′→3′)	Reverse (5′→3′)	Reference
*vasa*	CGTGATTCAGGTGACCCAGTT	GCCCGTGGTGTTCAGGAA	[30]
*cyp19a1a*	CAGAGAAGTTGGATGATGCTGACT	GCTCCCCGTGGTTCTGAGC	[31]
*foxl2a*	CCACCCACTCCTATGCCCTAT	GCCGACAGTCCTTTGACGTT	[31]
*zp3*	GAGTTGGTGGTGGTCAAAG	CATACTGTCCACCATACAGCC	[31]
*zar1*	CATCTCTGGAACCAATAAGGTG	CCACCCTGTACGGATTGAAC	[31]
*foxl2b*	CATTCTGACGCTCACCACCTT	CTTGTTGCGTCTGGAGAGGAA	[31]
*β-actin*	AATCCACGAGACCACCTTCAACT	TGATCTCTTTCTGCATTCTGTCG	[31]

**Table 3:** Growth performance of eels fed the experimental diets with different soy isoflavone contents for 30 weeks.

Parameters	SI0	SI2.5	SI5	SI7.5	SI10	*p*-Value
Initial weight (g/fish)	1.34 ± 0.10	1.33 ± 0.03	1.25 ± 0.03	1.23 ± 0.06	1.24 ± 0.03	0.524
Final weight (g/fish)	5.82 ± 0.41 ^b^	12.60 ± 3.14 ^a^	9.52 ± 0.79 ^ab^	7.59 ± 1.33 ^ab^	7.86 ± 0.43 ^ab^	0.043
Total length (cm)	20.0 ± 0.23 ^b^	25.3 ± 1.02 ^a^	25.5 ± 0.66 ^a^	22.9 ± 1.08 ^ab^	22.6 ± 0.44 ^ab^	0.003
Survival (%)	88.5 ± 1.75	90.8 ± 4.99	95.9 ± 1.60	85.3 ± 5.68	85.4 ± 1.18	0.386
WG (g/fish)	4.48 ± 0.48 ^b^	11.27 ± 3.1 ^a^	8.27 ± 0.77 ^ab^	6.36 ± 1.28 ^ab^	6.62 ± 0.42 ^ab^	0.045
SGR (%/day)	0.70 ± 0.06 ^b^	1.04 ± 0.12 ^a^	0.96 ± 0.04 ^ab^	0.85 ± 0.06 ^ab^	0.88 ± 0.02 ^ab^	0.040
CF (g/cm^3^)	0.12 ± 0.024	0.10 ± 0.002	0.10 ± 0.002	0.10 ± 0.002	0.10 ± 0.003	0.564
VSI (%)	4.27 ± 0.23	3.77 ± 0.24	3.95 ± 0.22	3.44 ± 0.21	3.43 ± 0.09	0.076
HSI (%)	1.39 ± 0.03 ^b^	1.37 ± 0.16 ^ab^	1.45 ± 0.08 ^a^	1.11 ± 0.11 ^ab^	0.96 ± 0.05 ^ab^	0.020

Values are mean ± standard error (SE; n = 3). Values with different superscript letters within a row are significantly different (*p* < 0.05), whereas the mean values in the same row without any superscripts are not different. WG, weight gain; SGR, specific growth rate; CF, condition factor; VSI, viscerosomatic index; HSI, hepatosomatic index.

**Table 4:** Chemical composition (%, wet weight) of the whole body of fish fed experimental diets with different soy isoflavone contents for 30 weeks.

Parameters	SI0	SI2.5	SI5	SI7.5	SI10	*p*-Value
Moisture	69.1 ± 0.23	68.6 ± 0.29	69.4 ± 0.21	69.9 ± 0.48	69.3 ± 0.20	0.125
Crude protein	16.4 ± 0.09 ^d^	18.1 ± 0.18 ^a^	17.6 ± 0.20 ^ab^	17.1 ± 0.14 ^bc^	16.7 ± 0.13 ^cd^	<0.001
Crude lipid	11.7 ± 0.15 ^a^	10.9 ± 0.08 ^bc^	10.4 ± 0.21 ^c^	11.2 ± 0.05 ^ab^	11.3 ± 0.18 ^ab^	0.001
Ash	2.2 ± 0.04	2.3 ± 0.04	2.1 ± 0.05	2.0 ± 0.10	2.1 ± 0.0	0.127

Values are mean ± SE (n = 3). Values with different superscript letters within a row are significantly different (*p* < 0.05), whereas the mean values in the same row without any superscripts are not different.

## Data Availability

Data are available upon reasonable request.

## References

[1] Ahn J.C., Chong W.S., Na J.H., Yun H.B., Shin K.J., Lee K.W., Park J.T. (2015). An evaluation of major nutrients of four farmed freshwater eel species (*Anguilla japonica, A. rostrata, A. bicolor pacifica* and *A. marmorata*). Korean J. Fish. Aquat. Sci..

[2] Hakoyama H., Faulks L., Rousseau Y., Kodama S., Okamoto C., Fujimori H., Sekino M. (2022). Japanese Eel, *Anguilla japonica*. Current Status of International Fishery Stocks in 2022.

[3] Hamidoghli A., Bae J., Won S., Lee S., Kim D.J., Bai S.C. (2019). A review on Japanese eel (*Anguilla japonica*) aquaculture, with special emphasis on nutrition. Rev. Fish. Sci. Aquac..

[4] Yuan Y., Yuan Y., Dai Y., Gong Y., Yuan Y. (2022). Development status and trends in the eel farming industry in Asia. N. Am. J. Aquac..

[5] Korean Statistical Information Service (KOSIS). https://kosis.kr/statHtml/statHtml.do?sso=ok&returnurl=https%3A%2F%2Fkosis.kr%3A443%2FstatHtml%2FstatHtml.do%3Flist_id%3DK2_7%26obj_var_id%3D%26seqNo%3D%26tblId%3DDT_1EW0004%26vw_cd%3DMT_ZTITLE%26orgId%3D101%26path%3D%252FstatisticsList%252FstatisticsListIndex.do%26conn_path%3DMT_ZTITLE%26itm_id%3D%26lang_mode%3Dko%26scrId%3D%26.

[6] Higuchi M., Mekuchi M., Hano T., Imaizumi H. (2019). Trans-omics analyses revealed differences in hormonal and nutritional status between wild and cultured female Japanese eel (*Anguilla japonica*). PLoS ONE.

[7] Inaba H., Iwata Y., Suzuki T., Horiuchi M., Surugaya R., Ijiri S., Uchiyama A., Takano R., Hara S., Yazawa T. (2023). Induce feminization of Japanese eel (*Anguilla japonica*). Int. J. Mol. Sci..

[8] Ohta H., Kagawa H., Tanaka H., Okuzawa K., Iinuma N., Hirose K. (1997). Artificial induction of maturation and fertilization in the Japanese eel, *Anguilla japonica*. Fish Physiol. Biochem..

[9] Chai Y., Tosaka R., Abe T., Sago K., Sago Y., Hatanaka E., Ijiri S., Adachi S. (2010). The relationship between the developmental stage of oocytes in various seasons and the quality of the egg obtained by artificial maturation in the feminized Japanese eel *Anguilla japonica*. Aquac. Sci..

[10] Chiba H., Iwatsuki K., Hayami K., Yamauchi K. (1993). Effects of dietary Estradiol-17β on feminization, growth and body composition in the Japanese eel (*Anguilla japonica*). Comp. Biochem. Physiol..

[11] Satoh H., Nimura Y., Hibiya T. (1992). Sex control of the Japanese eel by an estrogen (DES-Na) in feed. Nippon Suisan Gakkaishi.

[12] Yokouchi K., Kaneko Y., Kaifu K., Aoyama J., Uchida K., Tsukamoto K. (2014). Demographic survey of the yellow-phase Japanese eel in Japan. Fish. Sci..

[13] Hwang J.A., Park J., Kim J.E., Lee J.H., Kim H.S. (2022). Estradiol-17β levels as a tool for sex determination in Farmed *Anguilla japonica*. Biochem. Biophys. Res. Commun..

[14] Tzchori I., Degani G., Elisha R., Eliyahu R., Hurvitz A., Vaya J., Moav B. (2004). The influence of phytoestrogens and oestradiol-17 β on growth and sex determination in the European eel (*Anguilla anguilla*). Aquac. Res..

[15] Kim D.J., Lee B.I., Kim K.K., Kim E.O., Son M.H., Seong K.B. (2013). Effects of Estradiol-17β on the feminization of Japanase eel, *Anguilla japonica*. J. Life Sci..

[16] Furuita H., Ohta H., Unuma T., Tanaka H., Kagawa H., Suzuki N., Yamamoto T. (2003). Biochemical composition of eggs in relation to egg quality in the Japanese eel, *Anguilla japonica*. Fish Physiol. Biochem..

[17] El-Sayed A.F.M., Abdel-Aziz E.S.H., Abdel-Ghani H.M. (2012). Effects of phytoestrogens on sex reversal of Nile tilapia (*Oreochromis niloticus*) larvae fed diets treated with 17α-Methyltestosterone. Aquaculture.

[18] Pelissero C., Le Menn F., Kaushick S. (1991). Estrogenic effect of dietary soya bean meal on vitellogenesis in cultured Siberian sturgeon *Acipenser baeri*. Gen. Comp. Endocrinol..

[19] Francis G., Makkar H.P.S., Becker K. (2001). Antinutritional factors present in plant-derived alternate fish feed ingredients and their effects in fish. Aquaculture.

[20] Farooq S., Bhat N., Dar S.A., Malik M.A. (2025). Phytoestrogens in aquaculture: Friend or foe to fish growth and reproductive health?. Blue Biotechnol..

[21] Pastore M.R., Negrato E., Poltronieri C., Barion G., Messina M., Tulli F., Ballarin C., Maccatrozzo L., Radaelli G., Bertotto D. (2018). Effects of dietary soy isoflavones on estrogenic activity, cortisol level, health and growth in rainbow trout, *Oncorhynchus mykiss*. Aquac. Res..

[22] He L., Wang H., Li E., Huang Q., Wang X., Qiao F., Qin C., Qin J., Chen L. (2024). Effects of soy isoflavones on growth performance, antioxidant capacity, non-specific immunity and lipid metabolism of juvenile Chinese mitten crab, *Eriocheir sinensis*. Aquaculture.

[23] DiMaggio M.A., Kenter L.W., Breton T.S., Berlinsky D.L. (2016). Effects of dietary genistein administration on growth, survival and sex determination in southern flounder, *Paralichthys lethostigma*. Aquac. Res..

[24] Kiparissis Y., Balch G.C., Metcalfe T.L., Metcalfe C.D. (2003). Effects of the isoflavones genistein and equol on the gonadal development of Japanese medaka (*Oryzias latipes*). Environ. Health Perspect..

[25] Fajkowska M., Adamek-Urbańska D., Ostaszewska T., Szczepkowski M., Rzepkowska M. (2021). Effect of genistein, daidzein and coumestrol on sex-related genes expression in Russian sturgeon (*Acipenser gueldenstaedtii*). Aquaculture.

[26] Turan F., Yigitarslan K.D. (2019). Effect of immersion treatment of soybean isoflavones extract on sex reversal in the rainbow trout (*Oncorhynchus mykiss*, Walbaum, 1792). Biharean Biol..

[27] Hwang J.A., Park J.S., Jeong H.S., Hwang S.D. (2024). Influence of 17α-methyltestosterone on morphological deformities and pigmentation development in juvenile Japanese eels, *Anguilla japonica*. Animals.

[28] Association of Official Analytical Chemists (1990). Official Methods of Analysis.

[29] Jeong H.S., Kim J., Olowe O.S., Cho S.H. (2022). Dietary optimum inclusion level of jack mackerel meal for olive flounder (*Paralichthys olivaceus*, Temminck & Schlegel, 1846). Aquaculture.

[30] Jeng S.R., Wu G.C., Yueh W.S., Kuo S.F., Dufour S., Chang C.F. (2018). Gonadal development and expression of sex-specific genes during sex differentiation in the Japanese eel. Gen. Comp. Endocrinol..

[31] Horiuchi M., Hagihara S., Kume M., Chushi D., Hasegawa Y., Itakura H., Yamashita Y., Adachi S., Ijiri S. (2022). Morphological and molecular gonadal sex differentiation in the wild Japanese eel *Anguilla japonica*. Cells.

[32] Kim D.J., Lee N.S., Kim S.K., Lee B.I., Seong K.B., Kim K.K. (2013). Effects of water temperature and estradiol-17b on the sex ratio and growth of the japanese eel *Anguilla japonica*. J. Life Sci..

[33] Mai K., Zhang Y., Chen W., Xu W., Ai Q., Zhang W. (2012). Effects of dietary soy isoflavones on feed intake, growth performance and digestibility in juvenile Japanese flounder (*Paralichthys olivaceus*). J. Ocean. Univ. China.

[34] Gu M., Gu J.N., Penn M., Bakke A.M., Lein I., Krogdahl Å. (2015). Effects of diet supplementation of soya-saponins, isoflavones and phytosterols on Atlantic salmon (*Salmo salar*, L.) fry fed from start-feeding. Aquac. Nutr..

[35] Cao S., Xiong D., Luo W., Tang J., Qu F., Zhou Y., He Z., Xie S., Liu Z. (2020). Effects of dietary soy isoflavones on growth, antioxidant status, immune response and resistance of juvenile grass carp (*Ctenopharyngodon idella*) to *Aeromonas hydrophila* challenge. Aquac. Res..

[36] Zhou C., Lin H., Ge X., Niu J., Wang J., Wang Y., Chen L., Huang Z., Yu W., Tan X. (2015). The Effects of dietary soybean isoflavones on growth, innate immune responses, hepatic antioxidant abilities and disease resistance of juvenile golden pompano *Trachinotus ovatus*. Fish Shellfish Immunol..

[37] Hu Y., Zhang J., Xue J., Chu W., Hu Y. (2021). Effects of dietary soy isoflavone and soy saponin on growth performance, intestinal structure, intestinal immunity and gut microbiota community on rice field eel (*Monopterus albus*). Aquaculture.

[38] Jeong H.S., Cho S.H., Lee K.W. (2020). Dietary substitution effect of *Undaria pinnatifida* with onion extract byproduct on growth, chemical composition and air exposure stress of juvenile abalone (*Haliotis discus*, Reeve 1846). Aquaculture.

[39] Dai Q., Cho S.H. (2022). Dietary inclusion effect of citrus peel by-product as an additive on the growth performance, body composition, and various stress resistance of juvenile abalone (*Haliotis discus*) compared to ethoxyquin. Aquac. Rep..

[40] Heinsbroek L.T.N., Van Hooff P.L.A., Swinkels W., Tanck M.W.T., Schrama J.W., Verreth J.A.J. (2007). Effects of feed composition on life history developments in feed intake, metabolism, growth and body composition of European eel, *Anguilla anguilla*. Aquaculture.

[41] Ramseyer L.J. (2002). Predicting whole-fish nitrogen content from fish wet weight using regression analysis. N. Am. J. Aquac..

[42] Chatzifotis S., Panagiotidou M., Papaioannou N., Pavlidis M., Nengas I., Mylonas C. (2010). Effect of dietary lipid levels on growth, feed utilization, body composition and serum metabolites of meagre (*Argyrosomus regius*) juveniles. Aquaculture.

[43] Tesch F.W. (2003). The Eel.

[44] Uchida D., Yamashita M., Kitano T., Iguchi T. (2004). An aromatase inhibitor or high water temperature induce oocyte apoptosis and depletion of P450 aromatase activity in the gonads of genetic female zebrafish during sex-reversal. Comp. Biochem. Physiol. A Mol. Integr. Physiol..

[45] Guerrero-Estévez S., Moreno-Mendoza N. (2012). Gonadal morphogenesis and sex differentiation in the viviparous fish *Chapalichthys encaustus* (Teleostei, Cyprinodontiformes, Goodeidae). J. Fish Biol..

[46] Karube M., Fernandino J.I., Strobl M.P., Strussmann C.A., Yoshizaki G., Somoza G.M., Patino R. (2007). Characterization and expression profile of the ovarian cytochrome p-450 aromatase (cyp19A1) gene during thermolabile sex determination in pejerrey, *Odontesthes bonariensis*. J. Exp. Zool. A Ecol. Genet. Physiol..

[47] Robledo D., Ribas L., Cal R., Sánchez L., Piferrer F., Martínez P., Viñas A. (2015). Gene expression analysis at the onset of sex differentiation in turbot (*Scophthalmus maximus)*. BMC Genom..

[48] Inaba H., Hara S., Horiuchi M., Ijiri S., Kitano T. (2021). Gonadal expression profiles of sex-specific genes during early sexual differentiation in Japanese eel *Anguilla japonica*. Fish. Sci..

[49] Yamaguchi T., Yamaguchi S., Hirai T., Kitano T. (2007). Follicle-stimulating hormone signaling and Foxl_2_ are involved in transcriptional regulation of aromatase gene during gonadal sex differentiation in Japanese flounder, *Paralichthys olivaceus*. Biochem. Biophys. Res. Commun..

[50] Geffroy B., Guilbaud F., Amilhat E., Beaulaton L., Vignon M., Huchet E., Rives J., Bobe J., Fostier A., Guiguen Y. (2016). Sexually dimorphic gene expressions in eels: Useful markers for early sex assessment in a conservation context. Sci. Rep..

[51] Geffroy B., Bardonnet A. (2016). Sex differentiation and sex determination in eel: Consequences for management. Fish Fish..

[52] Amin E.M. (1997). Gonad differentiation and early gonadal development of the European eel *Anguilla anguilla* L. in Egyptian waters. Arab. Gulf J. Sci. Res..

[53] Krueger W.H., Oliveira K. (1999). Evidence for environmental sex determination in the American eel, *Anguilla rostrata*. Environ. Biol. Fishes.

